# Cost Efficiency of fMRI Studies Using Resting‐State Vs. Task‐Based Functional Connectivity

**DOI:** 10.1002/hbm.70260

**Published:** 2025-06-21

**Authors:** Xinzhi Zhang, Leslie A. Hulvershorn, Todd Constable, Yize Zhao, Selena Wang

**Affiliations:** ^1^ Department of Biostatistics Yale School of Public Health New Haven Connecticut USA; ^2^ Department of Psychiatry Indiana University School of Medicine Indianapolis Indiana USA; ^3^ Department of Radiology and Biomedical Imaging Yale School of Medicine, Yale University New Haven Connecticut USA; ^4^ Department of Biostatistics and Health Data Science Indiana University School of Medicine Indianapolis Indiana USA

## Abstract

We investigate whether and how we can improve the cost efficiency of neuroimaging studies with well‐tailored fMRI tasks. The comparative study is conducted using a novel network science‐driven Bayesian connectome‐based predictive method, which incorporates network theories in model building and substantially improves precision and robustness in imaging biomarker detection. The robustness of the method lays the foundation for identifying predictive power differentials across fMRI task conditions if such differences exist. When applied to a clinically heterogeneous transdiagnostic cohort, we find shared and distinct functional fingerprints of neuropsychological outcomes across seven fMRI conditions. For example, the emotional N‐back memory task is found to be less optimal for negative emotion outcomes, and the gradual‐onset continuous performance task is found to have stronger links with sensitivity and sociability outcomes than with cognitive control outcomes. Together, our results show that there are unique optimal pairings of task‐based fMRI conditions and neuropsychological outcomes that should not be ignored when designing well‐powered neuroimaging studies.

## Graphical abstract

We investigate whether and how we can improve the cost efficiency of neuroimaging studies with well‐tailored fMRI tasks. Our results show that there are unique optimal pairings of task‐based fMRI conditions and neuropsychological outcomes that should not be ignored when designing well‐powered neuroimaging studies.

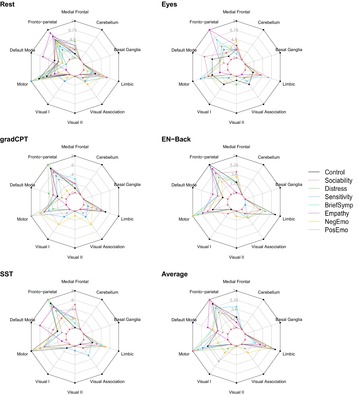

## 1 Introduction

Recent advances in functional magnetic resonance imaging (fMRI) technologies allow quantification of regional pairwise co‐activations across the brain, known as the functional connectome (FC). Association‐based analyses allow identification of brain regions with significant links to individual outcomes (e.g., Marek et al. 2019; Satterthwaite et al. 2015). Meanwhile, the rise of precision medicine devising patient‐tailored treatment plans encourages a shift in methods from association analyses to individual‐level connectome‐based predictive modeling (CPM, Finn et al. 2015; Shen et al. 2017). These models are used to recognize and select meaningful patterns from functional connectivity that explain differences across individuals for a range of cognitive and behavioral variants and disease outcomes.

A pervasive challenge in connectome‐behavior linking is to achieve maximum study predictive power and reproducibility given limited resources, e.g., cost of obtaining fMRI data per scan and person. In many fMRI studies, resting‐state data is the default condition for linking functional connectomes with behaviors, perhaps because the resting‐state acquisition protocol is easier to replicate and compare across studies. However, literature shows that cognitive tasks can amplify differences across individuals in connectivity that are relevant for explaining differences in various behavioral outcomes (Chen et al. 2023; Finn 2021; Finn et al. 2017). Resting‐state data is perhaps the worst data to use for building CPMs (Finn 2021; Zhao et al. 2023). Task‐based fMRI has been shown to outperform resting‐state fMRI in predictive modeling, likely due to its ability to directly engage neural circuits associated with specific cognitive functions, thereby capturing more behaviorally relevant information (Sripada et al. 2020). Unlike resting‐state fMRI, which relies on spontaneous fluctuations, task‐based paradigms elicit structured brain responses that better correlate with cognitive performance (Zhao et al. 2023). Studies demonstrate that activation patterns from cognitively demanding tasks, such as the N‐back task, strongly predict general cognitive ability, surpassing resting‐state functional connectivity (Sripada et al. 2020). Additionally, task‐induced brain states amplify individual differences, improving the prediction of traits (Greene et al. 2018).

Tasks performed during fMRI scanning sessions are often designed to stimulate a wide range of cognitive, emotional, and psychological processes that perturb brain circuits in unique ways. It is unlikely that one single task is ideal to differentiate individuals across all dimensions of outcomes. Different types of task fMRI tend to show varying strengths of predictive power for different behaviors. This differential in predictive power means that there is untapped potential to maximize the return for scientific investment with a given fMRI scan time and sample size by tailoring the fMRI task during scanning to achieve the most predictive power for addressing a particular research question in a specific clinical setting. Indeed, researchers have begun to investigate this possibility. For example, in schizophrenia research, Gur et al. (2014) find that emotion recognition tasks yield higher diagnostic accuracy compared to attention tasks. In the domain of anxiety disorders, Etkin and Wager (2007) show that certain tasks are more effective for distinguishing individuals for anxiety disorders related propensities. Regarding depression, Fitzgerald et al. (2008) find that more complex cognitive tasks, such as the Emotional N‐back (EN‐back) memory task, are more effective in distinguishing patients from healthy controls. Additionally, Kaiser et al. (2015) demonstrate that different reward‐processing tasks can reveal distinct neural biomarkers for depression. These studies collectively illustrate how task‐specific fMRI data can be used for certain types of outcomes as different behavioral categories may be more indicative of certain conditions under specific tasks (Wolfers et al. 2015). This underscores the necessity of carefully selecting and designing task‐based fMRI conditions to optimize their predictive power for specific behaviors under investigation.

To systematically evaluate the cost efficiency of task versus resting‐state fMRI studies, we investigate the efficacy of seven task and resting conditions and their associated brain circuits perturbation that may be beneficial to investigate a wide range of behavioral, emotional, and psychological outcomes. The cost of fMRI studies—due to the need for specialized equipment, trained personnel, and substantial scanning time—is quite high. Therefore, optimizing cost efficiency is essential in ensuring that fMRI research remains feasible and sustainable, particularly when scaling studies for larger populations. By investigating the efficacy of different fMRI conditions, we aim to identify those that provide the highest predictive power for behavioral outcomes relative to their cost. This would help maximize the value of fMRI research, ensuring that resources are used efficiently while maintaining scientific rigor. Furthermore, the cost efficiency of these methods is particularly crucial in clinical research settings, where resource allocation can be limited, and prioritizing the most effective and affordable methods is necessary.

Our study uniquely contributes to existing literature (1) by investigating this issue in a clinically heterogeneous cohort, the transdiagnostic dataset (Greene et al. 2022) spanning healthy individuals and those with one or more psychiatric disorders and (2) by fitting a novel Bayesian generative model called LatentSNA (Wang et al. 2023) that has been proven to bypass lower statistical power and replicability issues often found with existing methods. The transdiagnostic dataset is beneficial for investigating the unique fit of task‐fMRI to behavioral outcomes because the study participants have heterogeneous functional connectivity profiles given their diverse clinical diagnoses. Different fMRI tasks may invoke different excitatory patterns with participants with different clinical diagnoses. The heterogeneity would maximize the predictive power differential if such differences exist.

Our network science–driven Bayesian generative model offers several advantages for investigating differences in predictive power across resting‐ and task‐fMRIs for various individual outcomes. First, our model bypasses the lack of power found in existing fMRI‐based predictive models (Wang et al. 2023). Second, the joint modeling of brain and behavior in LatentSNA strengthens connectivity signals by allowing the true connectivity and internalizing signals to mutually inform each other. Third, our framework provides uncertainty quantification for both the connectome/behavior state and future outcome prediction, which is crucial for a reproducibility‐focused design in model building. Fourth, compared with existing network neuroscience models, our model incorporates network theories (Sweet and Wang 2024) in model building (versus relying on graph theory metrics for model interpretation), drawing on insights regarding the universality of the communicative structures of real‐world networks (Barabási 2013). Shared network architectures (Barabási 2013; Wohlgemuth 2012) inform us with a universal set of mathematical and statistical instruments for network generation. Overall, the model demonstrates substantially improved precision and robustness in imaging biomarker detection.

In this paper, we compare the performance of different types of resting‐ and task‐based fMRI in their capability to predict various neuropsychological measures and investigate differences in identified neuroimaging biomarkers. We define a neuroimaging biomarker as a measurable regional indicator that reflects individual differences in neuropsychological outcomes, disease processes, or abnormal functionalities (Kyle and Tavel 2010). Specifically, these neuroimaging biomarkers are collections of brain regions associated with specific behaviors. In doing so, we seek to uncover the unique neural signatures manifested through different task fMRIs that are linked with specific neuropsychological outcomes. Through this comprehensive approach, we strive to contribute to scientific discussions around the cost efficiency of fMRI studies to achieve maximum predictability and robustness and to highlight the unique benefits of well‐designed task fMRI studies for investigating complex neuropsychological outcomes.

## 2 Material and Methods

### 2.1 The Transdiagnostic Dataset

**FIGURE 1:**
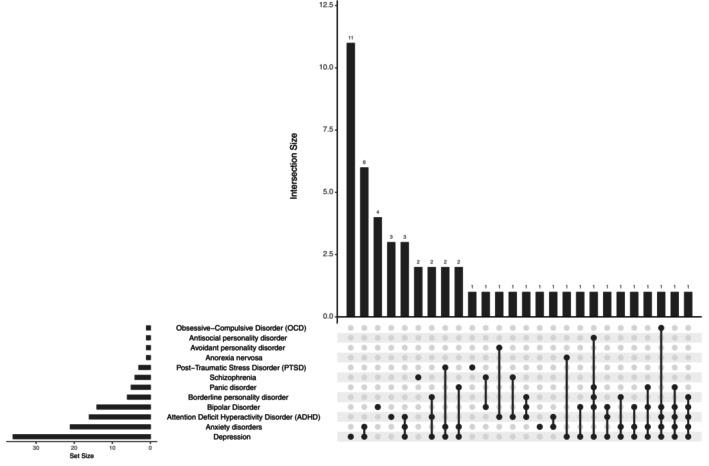
Comorbidity patterns among psychiatric disorders. This UpSet plot illustrates our dataset's frequency and co‐occurrence of mental health diagnoses. The left axis shows the total number of patients for each disorder, while the main plot displays the size and composition of various comorbidity combinations. Each column represents a unique combination of disorders, with connected dots below indicating the specific disorders involved. The height of each bar reflects the number of patients with that exact combination of diagnoses.

We used the transdiagnostic dataset collected at Yale University (Greene et al. 2022). We included 190 total participants with 75 females and 54 males. The participants' ages ranged from 19 to 67 years. Among them, 76 were not diagnosed with any mental health condition; 53 had at least one mental health disorder, such as depression, Attention Deficit Hyperactivity Disorder (ADHD), psychotic disorder, schizophrenia, or Post‐Traumatic Stress Disorder (PTSD). Of the 53 participants with psychiatric disorders, 29 had a single diagnosis; 11 patients had 2 diagnoses; 8 patients had 3 diagnoses; 3 had 4 diagnoses; and 2 patients had 5 diagnoses. The co‐occurrence of these mental health diseases was shown in Figure 1. The number of patients for each disorder was detailed in Table S1.

With the transdiagnostic dataset, there were seven resting/task conditions for fMRI data, including an average condition used as a comparison. In addition, participants underwent a structured diagnostic interview and completed a battery of scales derived from validated neuropsychological self‐report measures outlined below. Together with each participant, we had both fMRI data from various conditions and a range of behavioral measures.

#### 2.1.1 Neuropsychological Measures

As mentioned before, each participant was asked to complete a set of neuropsychological measures, which could be grouped into eight categories, each measuring a distinct latent construct based on the design of the associated survey. We assumed unidimensionality of each category of neuropsychological measures, though a more comprehensive analysis was warranted to confirm its efficacy and validity; assessing the dimensionality of these surveys was outside the scope of the current study. This categorization allowed us to perform a more targeted analysis of the relationships between brain activity and neuropsychological outcome. The detailed description of each category was as follows.

- **Negative emotional spectrum (NegEmo)**: The NegEmo category assessed negative emotional experiences. This category focused on measuring different facets of emotional distress and unpleasant mood states. The NegEmo category was measured by the Positive and Negative Affect Schedule—Expanded Version (Watson and Clark 1994) including negative affect, fear, sadness, guilt, hostility, shyness, and fatigue.
- **Positive emotional spectrum (PosEmo)**: The PosEmo category measured pleasant emotional states, giving a complete picture of a person's positive feelings and experiences. This category looked at different types of good moods and emotional well‐being. It contained six variables: positive affect, joviality, assurance, attentiveness, serenity, and surprise. It was also from the positive and negative affect schedule—expanded version (Watson and Clark 1994).
- **Empathy engagement scale (Empathy)**: The Empathy category evaluated a person's ability to understand and feel other people's emotions. This category examined the different ways that individuals relate to and demonstrate empathy. It contained four variables: perspective taking, fantasy, empathic concern, and personal distress. This category came from the interpersonal reactivity index (Davis 1983).
- **Emotional distress spectrum (Distress)**: The Distress category measured emotional distress and negative affective states in adults. This category captured how people experience and express psychological discomfort and unpleasant emotions in response to challenging situations. It contained four measures: fear, sadness, discomfort, and frustration. It was derived from the adult temperament questionnaire (Evans and Rothbart 2007).
- **Sociability and vitality scale (Sociability)**: The Sociability category captured the tendency to seek out and enjoy social interactions, as well as the experience of high‐energy positive emotions. It included three key variables derived from the surgency part of the adult temperament questionnaire (ATQ) (Evans and Rothbart 2007): sociability, positive affect, and high‐intensity pleasure.
- **Self‐regulation control measures (Control)**: The Control category assessed various aspects of an individual's ability to manage their thoughts, emotions, and behaviors. This category captured the capacity for self‐regulation and effortful control in adults. It contained three variables from the adult temperament questionnaire (Evans and Rothbart 2007): Attentional Control, Inhibitory Control, and Activation Control.
- **Sensory and emotional awareness scale (Sensitivity)**: The Sensitivity category assessed a person's sensitivity to both internal and external stimuli and their level of perceptual awareness. The range and depth of an adult's sensory processing and associative tendencies were summarized in this category. It included four measures from the adult temperament questionnaire (Evans and Rothbart 2007): neutral perceptual sensitivity, affective perceptual sensitivity, and associative sensitivity.
- **Psychological symptoms inventory (BriefSymp)**: The BriefSymp category measured a range of psychological symptoms and psychiatric disorders (Derogatis and Melisaratos 1983). The BriefSymp category contained nine variables: somatization, obsession‐compulsion, interpersonal sensitivity, depression, anxiety, hostility, phobic anxiety, paranoid ideation, and psychoticism.

#### 2.1.2 Functional Connectivity Data

In our study, we utilized functional connectivity (FC) data derived from seven different fMRI conditions: two resting‐state conditions, four task‐based conditions, and one average condition. Participants were asked to relax with their eyes open, not engaging in any specific task during resting‐state conditions. Participants were asked to perform specific cognitive or emotional tasks that activated targeted brain regions during task conditions (Shah et al. 2010). These fMRI conditions allowed us to explore differences between fMRI conditions and their predictability of different neuropsychological measures. A detailed description of four task‐based fMRI conditions was as follows:

- **Emotional N‐back task (EN‐back)**: The EN‐back task condition was designed to measure working memory and emotional processing. Participants were asked to identify if an image was the same or different from the one that appeared earlier (Conley et al. 2018; Gevins et al. 1990; Rosenberg et al. 2015; Tottenham et al. 2009).
- **Stop‐signal task (SST)**: The SST task engaged cognitive systems, specifically working memory. In this task, participants were instructed to respond as quickly as possible to a go stimulus unless a stop signal appeared after a variable delay, at which point they were to attempt to withhold their response (Verbruggen et al. 2008).
- **Reading the mind in the eyes task (Eyes)**: The Eyes task involved cognitive systems and exercised cognitive control. Participants were to identify the direction of an arrow stimulus and withhold their response if the arrow turned blue (Cohen et al. 1997).
- **Gradual‐onset continuous performance task (gradCPT)**: The gradCPT task tapped into cognitive systems emphasizing attention. Participants were asked to press a button when they saw images of cities but were asked to refrain from responding when they saw images of mountains (Rosenberg et al. 2013).

### 2.2 MRI Data Acquisition and Pre‐Processing

Functional Magnetic Resonance Imaging (fMRI) data were acquired using a Siemens Prisma 3T scanner. The repetition time (TR) was set to 720 ms to enable rapid data acquisition. An echo time (TE) of 33.1 ms was used to optimize signal sensitivity. The flip angle was adjusted to 52° to enhance image contrast. Each voxel measured 2.0 mm^3^, providing a balance between spatial resolution and signal‐to‐noise ratio. To accelerate data collection, a multiband acceleration factor of 8 was applied, allowing for the simultaneous acquisition of multiple slices.

The acquired data were preprocessed using BioImage Suite (Joshi et al. 2011), following established protocols (Greene et al. 2018; Horien et al. 2019). Slice timing correction was performed using SPM8 to account for differences in acquisition timing across slices (Horien et al. 2019). Motion correction was also conducted using SPM8, aligning all volumes to a reference image to reduce motion artifacts (Horien et al. 2019). Spatial normalization was applied by transforming the images into the Montreal Neurological Institute (MNI) template space through linear and nonlinear transformations (Horien et al. 2019). To mitigate physiological noise, nuisance signal regression was performed by removing mean signals from cerebrospinal fluid (CSF), white matter (WM), and the global signal (Horien et al. 2019). Temporal filtering was applied using a low‐pass Gaussian filter with a cutoff frequency of 0.12 Hz to reduce high‐frequency noise while preserving neural‐related signals (Horien et al. 2019). Horien et al. (2019) suggest excluding participants with a mean framewise displacement (FD) exceeding 0.1 mm to maintain data quality, and no participant was excluded from our study. Although we did not perform direct statistical comparisons of head motion between experimental conditions, strict motion thresholds were enforced to minimize motion‐related confounds (Horien et al. 2019).

To define brain regions, we employed the Shen‐268 atlas (Shen et al. 2010), which divided the brain into 268 distinct Regions of Interest (ROIs). Functional connectivity matrices were computed for each participant under different conditions, including both task‐based and resting‐state fMRI. This process yielded six 268 × 268 connectivity matrices per subject, where each matrix element represented the Pearson correlation coefficient between two brain nodes. These correlation values were then normalized using Fisher's *z*‐transformation to enhance statistical validity.

For network‐based analysis, we categorized the 268 ROIs into ten functional systems based on their anatomical and functional properties: Default Mode, Medial Frontal, Fronto‐parietal, Motor, Visual I, Visual II, Visual Association, Limbic, Basal Ganglia, and Cerebellum (Shen et al. 2010).

### 2.3 Methods

We used the Bayesian generative LatentSNA model to investigate differences in predictive power across task‐fMRIs for a range of individual outcomes. In this section, we provided an overview of the LatentSNA model, then detailed the procedures for calculating the prediction accuracy and the process for identifying neuroimaging biomarkers. To maximize replicability and robustness of the results, we employed independent replications of our model results at each behavior‐fMRI condition pairing via random sampling (replication robustness) with alternative neuropsychological measures (independent neuropsychological data robustness) and with alternative fMRI conditions (independent fMRI data robustness).

#### 2.3.1 Overview of LatentSNA Model

In a previous study (Wang et al. 2023), we had shown that by incorporating network theories in the data generation process and model building, we could substantially improve the predictability of an individual's psychological outcomes using fMRI data. This capability of our method allowed us to investigate effective differences between fMRI obtained under different task conditions. Satisfactory prediction accuracy in independent samples indicated a level of robustness of the model and served as an important basis for the comparative analysis. In addition, we identified the critical brain regions linked with neuropsychological outcomes. Lastly, the LatentSNA model allowed us to input multiple behavior indicators to precisely measure the corresponding latent construct.

In this section, we provided an overview of the method although due to space constraints, we could not list all mathematical details here; interested readers were referred to the methodology paper (Wang et al. 2023). The LatentSNA method proposed a generative statistical process in which each person's (V×V) connectivity matrix was reduced to a one‐dimensional latent position vector of dimension (V×1). This latent position vector captured the essential features of the individual's brain network connectivity in a lower‐dimensional space, which could then be integrated with neuropsychological behavior variables. Region‐specific connectivity information was then linked with neuropsychological measures to capture significant associations. Suppose we had V×V functional connectivity matrix $C_{j}$ as well as the P×1 behavioral vector data $y_{j}$ for subject *j*; *P* was the total number of variables measuring the unidimensional latent neuropsychological construct; In total we have N subjects. For each the connectivity between nodes *x* and *y*, x<y,x,y=1,2,…,V is modeled by

(1) $$c_{x,y,j}=d_{j}+\gamma_{x,j}\gamma_{y,j}+f_{x,y,j},\;\;f_{x,y,j}\overset{\mathrm{iid}}{∼}N\left(0,\tau^{2}\right)$$

where $c_{x,y,j}$ represented the connectivity between the brain nodes *x* and *y;* $d_{j}$ represented the intercept; $f_{x,y,j}$ was the error term, and $\tau^{2}$ was the error variance.

For the behavior component of the joint model, subject *j*'s response on variable *p* is modeled by

(2) $$b_{j,p}=e_{p}+\kappa_{j}+v_{j,p},\;\;v_{j,p}\overset{\mathrm{iid}}{∼}N\left(0,\sigma^{2}\right)$$

where $b_{j,p}$ represented the behavior variable *p* of subject *j*; $e_{p}$ represented the intercept; $v_{j,p}$ was the error term; and $\sigma^{2}$ was the error variance; $\kappa_{j}$ was the latent variable of the behavior.

The latent variables of connectivity and individual behavioral variables are linked in the following way:

(3) $${\left(\gamma_{1,j},\gamma_{2,j},\ldots ,\gamma_{V,j},\kappa_{j}\right)}^{T}\overset{\mathrm{iid}}{∼}\mathrm{MVN}\left(\left(\begin{array}{c} 0_{V}\\ 0_{1} \end{array}\right),\sum_{V+1}\right)$$

The model was estimated with a novel MCMC algorithm detailed in Wang et al. (2023). When fitting the LatentSNA model, we used 5000 burn‐in iterations and 15,000 MCMC samples. Our model, which incorporated one behavioral variable and functional connectivity data from a fMRI condition for 190 subjects, took approximately 10 h to fit using a single‐core processor and about 8 GB of memory. This timeframe and resource usage represented the computational requirements for fitting just one instance of our model. To avoid being stuck in the local optima, we fitted each model with 10 random parameter initializations and chose the most optimal model based on model fit in the test sample. The process was repeated 5 times for cross‐validation. To ensure fair comparison with competing methods, we used identical training and test samples across all methods. To assess convergence, we computed the Gelman‐Rubin diagnostic ($\hat{R}$) for each fMRI condition‐behavioral category pair across the covariance estimates of all brain regions, with 75% of pairs achieving a mean $\hat{R}<1.1$ when averaged across brain regions, indicating satisfactory convergence. The trace plot of the covariance estimate in Figure S1 demonstrated the convergence of our model.

#### 2.3.2 Predictive Robustness Analysis

To investigate differences in predictive power among different fMRI task conditions for a range of neuropsychological measures, we applied the LatentSNA method detailed above to predict each neuropsychological variable using fMRI data from distinct conditions: four task‐based conditions (Emotional N‐back task, Stop‐signal task, Reading the Mind in the Eyes task, and Gradual‐onset Continuous Performance Task), two resting‐state conditions, and one averaged condition. The Emotional N‐back task (EN‐back) measures working memory and emotional processing by asking participants to decide whether a face currently presented to them matches the one shown earlier (Conley et al. 2018; Gevins et al. 1990; Rosenberg et al. 2015; Tottenham et al. 2009). The Stop‐signal task (SST) assesses response inhibition, where participants are asked to respond to a go stimulus unless a stop signal appears, assessing their capability to suppress their response (Verbruggen et al. 2008). The Reading the mind in the eyes task (Eyes) evaluates social cognition by asking participants to infer mental states from eye expressions, which includes an arrow discrimination component that requires inhibitory control (Cohen et al. 1997). The Gradual‐onset Continuous Performance Task (gradCPT) measures sustained attention, asking participants to respond to city images while withholding responses to mountain images, engaging attentional control mechanisms (Rosenberg et al. 2013). We aimed to assess the optimality of each fMRI condition for different neuropsychological categories based on their predictive powers. In the following, we provided details about the procedures.

- Training sample selection. We randomly selected the training sample for the neuropsychological measures, which included 90% of the participants. The remaining 10% of the participants were reserved as the test sample. This same training and test sample split was used for all models, including the LatentSNA model and the five competing methods detailed below.
- Model training. We trained the LatentSNA model using the functional connectivity of all participants and neuropsychological measures from the training sample to predict the test sample's neuropsychological scores. The five competing methods were trained using the same input data.
- Cross‐validation. We repeated the previous two steps five times to perform 5‐fold cross‐validation for each connectivity and behavior combination.
- Accuracy measurement. We calculated the prediction accuracy for each model by calculating the correlation between the estimated behavior scores and the actual behavior scores in the test sample.
- Averaging accuracy across folds. We averaged the prediction accuracy across all folds to obtain a final accuracy measure for each model, allowing for a direct comparison between LatentSNA and the five competing methods.

**FIGURE 2:**
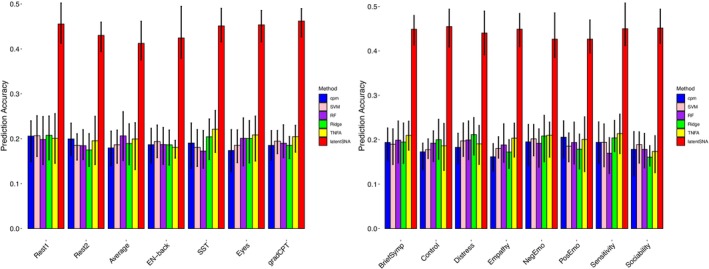
Distribution of prediction accuracy by task and behavioral category. The figure presents two bar plots summarizing prediction accuracy across different fMRI conditions and behavioral categories. Left plot: This plot presents the *averaged prediction accuracy across different behavioral outcomes*, demonstrating how well each fMRI condition predicts neuropsychological measures. Each bar corresponds to an fMRI condition (e.g., Rest1, Eyes, etc.), and the error bars indicate the interquartile range (25th–75th percentiles), quantifying variability in prediction performance. Right plot: This visualization depicts the *averaged prediction accuracy across different fMRI conditions*, summarizing the predictive performance of each neuropsychological measure (e.g., BriefSymp, Control, etc.) across all included fMRI conditions. The main bars represent the mean prediction accuracy, while the error bars indicate variability, providing insight into the consistency of predictive performance across different methods and conditions.

In our analyses, we compared the predication accuracy of our LatentSNA model against five existing methods, CPM (Finn et al. 2015; Shen et al. 2017), Ridge CPM (Rosenblatt et al. 2023), Tensor Network Factorization Analysis (TNFA, Zhang et al. 2019), Support Vector Machines (SVM, Hearst et al. 1998), and Random Forest (RF, Belgiu and Drăguţ 2016). We implemented CPM with NetworkToolbox (Christensen 2023); We first vectorized the upper triangular part (including the diagonal elements) of the symmetric *V* × *V* matrix into a $\frac{V^{2}+V}{2}\times 1$ vector through row‐wise concatenation, following established CPM protocols (Finn et al. 2015). This vectorization approach enables us to treat each connection as an independent predictor variable in subsequent analyses. Then we used a 0.001 threshold to identify significant connectivity edges as predictors. The threshold value of 0.001 was used methodological guidelines established in (Shen et al. 2017). This specific threshold value optimizes the trade‐off between type I error control and feature retention—it maintains sufficient stringency for multiple comparison correction while preserving edges with significant predictive utility. Our sensitivity analyses demonstrated that more liberal thresholds (e.g., 0.01) introduced excess noise into the feature set, while more conservative thresholds (e.g., 0.0001) eliminated potentially informative connectivity patterns. We implemented Ridge regression with ‘glmnet’ R package (Friedman et al. 2023); we used an L2 penalized linear regression and optimized the penalized parameter to handle high‐dimensional data. We implemented SVM with ‘e1071’ (Meyer et al. 2023), and we employed a linear kernel, selected features with correlations above 0.25 with the response, and tuned the cost parameter. We implemented the RF model with ‘ranger’ (Wright and Ziegler 2017) via ‘caret’ (Kuhn 2023). And we underwent a grid search for the optimal number of variables to split at each node (mtry), the splitting rule for tree size (splitrule), and the minimal node size (min.node.size). The number of decision trees used in the Random Forest algorithm is 500. We optimized each model on the training set and evaluated performance by correlating predicted values with observed values on the test set, allowing a thorough comparison of these techniques. For all methods, we used identical training and test sets to ensure fair comparison. The comparison of the prediction accuracy across all methods is shown in Figure 2.

To directly answer the question which fMRI conditions are optimal for investigating a particular type of neuropsychological measures, we conducted a series of regression analyses using the LatentSNA prediction results and summarized results in Table S2 (rest1 as reference condition). Additionally, we conducted a supplementary analysis using Rest2 as the reference condition; these results are summarized in Table S3. For each of the eight behavior categories (BriefSymp, NegEmo, PosEmo, Empathy, Distress, Sociability, Control, and Sensitivity), we ran a separate model. For each model, the dependent variable was the prediction accuracy for all indicators within the neuropsychological category. This means we analyzed how accurately we could predict all the indicators in a given category (e.g., all indicators of NegEmo) under different fMRI conditions. The predictors were dummy variables representing different fMRI conditions: Rest2, Average, EN‐back, SST, Eyes, and gradCPT. The Rest1 condition served as the reference, hence its absence from the predictor list. The mathematical form of the regression model for each neuropsychological category was:

(4) $$\begin{array}{c} \mathrm{PA}=\beta_{0}+\beta_{1}I\left(\text{Rest}2\right)+\beta_{2}I\left(\text{Average}\right)+\beta_{3}I\left(\mathrm{EN}-\text{back}\right)\\ +\beta_{4}I\left(\mathrm{SST}\right)+\beta_{5}I\left(\text{Eyes}\right)+\beta_{6}I\left(\text{gradCPT}\right) \end{array}$$

where PA represents the prediction accuracy for all indicators in the specific neuropsychological category being analyzed under different fMRI conditions; $I\left(\text{condition}\right)$ is the dummy variable for each fMRI condition; $\beta_{0}$ represents the baseline prediction accuracy under the Rest1 condition; and $\beta_{1}$ to $\beta_{6}$ represent the change in prediction accuracy for each respective fMRI condition compared to Rest1. This analysis, summarized in reveals how different fMRI task conditions influenced our ability to predict behavioral variables across various categories.

#### 2.3.3 Neuroimaging Biomarker Detection

In addition to comparing the predictive power of different task fMRI associated with each neuropsychological measure, we were also interested in the differences in identified neuroimaging biomarkers. To do this, we obtained region‐specific covariance parameters between behaviors and brain nodes by fitting the LatentSNA model with each category of neuropsychological measures and each fMRI condition. A high covariance/correlation value indicated a strong association between the neuropsychological measure and the regional functional connectivity data. The covariance estimate for each brain region was derived from the posterior mean. To validate our findings, each model underwent a 5‐fold cross‐validation using randomly chosen training samples, and the average covariance was calculated as the mean of the covariance estimates from the five folds. This approach allowed each model to determine the involvement level of functional biomarkers in a specific neuropsychological category.

After estimating the covariances between behavior categories and brain nodes, we selected the top 20 brain nodes, comprising 10 nodes with the smallest negative covariance values and 10 nodes with the largest positive covariance values. Using the Shen‐268 atlas, we mapped each brain node to the corresponding functional networks. This allowed us to investigate the comparable involvement of functional systems in each task fMRI–neuropsychology association analyses.

**FIGURE 3:**
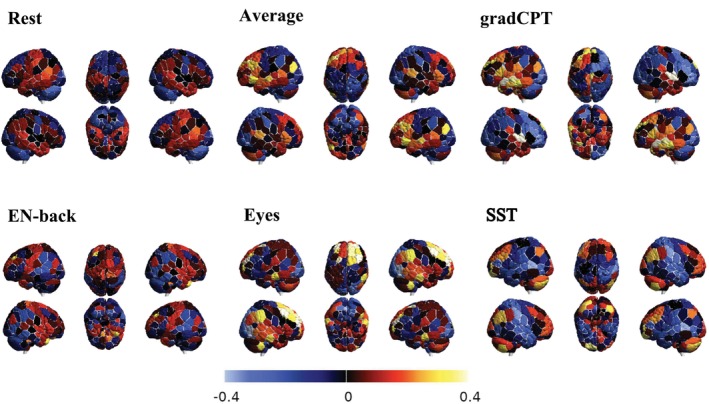
Covariance estimates between brain regions and sociability across various fMRI conditions. This figure visualizes the covariance estimates of 268 brain nodes across different fMRI conditions: Rest, Average, gradCPT, EN‐back, Eyes, and SST. The color scale indicates the magnitude of covariance, ranging from −0.4 (blue) to 0.4 (yellow). A lighter color indicates a higher absolute value of covariance. The visualization is generated using the ROI drawing function in BrainNetViewer.

**FIGURE 4:**
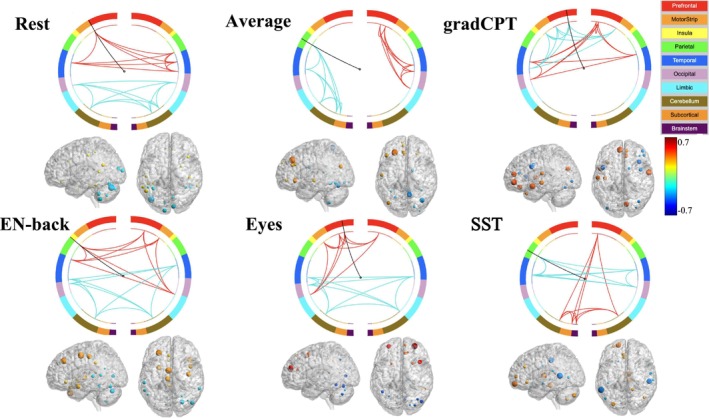
Circle plot and brain plot corresponding to top brain nodes for each task condition. The Circle plot demonstrates the functional connectivity patterns of brain nodes, specifically highlighting the top 5 positive covariance nodes (visualized in red) and top 5 negative covariance nodes (visualized in blue) for Sociability across distinct fMRI conditions. The complementary brain plot illustrates the anatomical positions of the top 20 brain nodes. Node size quantifies the absolute magnitude of the covariance estimate, while node color encodes the directionality and magnitude of covariance, with blue indicating negative values and red indicating positive values.

We characterized the functional biomarkers associated with distinct behavioral categories through multiple complementary visualization approaches. For the comparative analysis of behavioral categories, we employed a spider plot visualization framework implemented via the radarchart function from the R package fmsb (Nakazawa 2013). This visualization technique facilitated the examination of similarities and differences across behavioral categories under varying fMRI conditions. Each spider plot represented the distribution of functional biomarkers specific to a behavioral category, with inputs derived from the frequency distribution of functional labels among the top 20 brain nodes across all fMRI conditions. To comprehensively examine whole‐brain covariance patterns, we implemented two additional visualization methodologies. The Brain plot (Figure 3) was generated using BrainNet software by incorporating the Shen‐268 atlas surface and volumetric files. Covariance estimates across all fMRI conditions were visualized using BrainNet's region‐of‐interest (ROI) delineation functionality, enabling comprehensive mapping of whole‐brain connectivity patterns. Additionally, we employed the BioImage Suite Web platform Papademetris et al. (2006) to generate circle plots (Figure 4), which provide a complementary visualization of the connectivity relationships between functional biomarkers.

## 3 Results

Our Results section presented a systematic investigation of brain‐behavior relationships through three complementary analytical frameworks. In Section 3.1, we quantified the predictive performance of distinct fMRI conditions across behavioral categories, establishing condition‐specific prediction accuracies to identify the optimal fMRI condition for each behavioral category. In Section 3.2, we characterized the functional network architectures (fingerprints) associated with distinct behavioral categories, revealing both shared and unique patterns of brain connectivity. In Section 3.3, we conducted a detailed comparative analysis of functional network engagement patterns across different fMRI conditions.

### 3.1 Similarities and Differences Exist in the Predicability of fMRI Task/Resting Conditions

Satisfactory prediction accuracy from latentSNA was found across seven fMRI conditions and eight behavioral categories when compared with other methods. Figure 2 (left) shows the averaged prediction accuracy across behavioral outcomes, demonstrating how well each fMRI condition predicts neuropsychological measures. We systematically implemented the latentSNA model and alternative competing methods across behavioral variables and fMRI conditions. The five competing methods include CPM (Finn et al. 2015; Shen et al. 2017), Ridge CPM (Rosenblatt et al. 2023), Tensor Network Factorization Analysis (TNFA, Zhang et al. 2019), Support Vector Machines (SVM, Hearst et al. 1998), and Random Forest (RF, Belgiu and Drăguţ 2016). With 39 behavioral variables (see details in Methods section) and 7 fMRI conditions (see details in Methods section), we fitted LatentSNA to each of the 39×7 combinations. In Figure 2 (left), we calculated the average prediction accuracy associated with each of the fMRI conditions (Rest1, Rest2, Average, EN‐back, SST, Eyes, gradCPT), where each bar quantifies the predictive capacity using brain connectivity data derived from a specific fMRI condition when applied across the entire spectrum of behavioral variables.

Figure 2 (right) shows the averaged prediction accuracy across fMRI conditions, summarizing the predictive performance for each behavioral category. We stratified the prediction accuracy by behavioral category. The 39 behavioral variables were organized into 8 distinct categories (BriefSymp, Control, Distress, Empathy, NegEmo, PosEmo, Sensitivity, Sociability), where each bar represents the average predictive accuracy for variables within a specific category, utilizing data integrated across all fMRI conditions. By comparing predictability across behaviors and fMRI conditions, we convey two insights. First, predictive capacity varies across fMRI conditions and behavioral categories. Second, there are substantial methodological variations in predictive performance, with latentSNA consistently showing superior accuracy compared to alternative approaches. This consistent performance advantage of LatentSNA provides the empirical justification for our utilization of this method in all subsequent analyses presented in this study.

The following analysis focused on the LatentSNA method. When comparing across fMRI conditions, the average correlation between predicted and observed neuropsychological measures was approximately 0.45, with variation across fMRI conditions. The Average condition had a mean prediction accuracy of 0.41 (SD = 0.12), which was lower than Eyes (M = 0.45, SD = 0.08), gradCPT (M = 0.46, SD = 0.09), Rest1 (M = 0.46, SD = 0.08), and SST (M = 0.45, SD = 0.09). This suggested that important features might be lost when averaging across conditions, leading to decreased accuracy across a range of measures. In addition, EN‐back exhibited greater variability in prediction accuracy (M = 0.42, SD = 0.14), compared to resting‐state conditions (Rest1: SD = 0.08, Rest2: SD = 0.07). Several conditions demonstrated comparable predictive capabilities. When comparing the two resting‐state conditions, Rest1 had a slightly higher prediction accuracy than Rest2 (M = 0.46 vs. M = 0.43). This suggested that while both resting‐state conditions provided relatively stable predictive performance, Rest1 may offer a slightly stronger signal for neuropsychological measures. However, compared to task‐based conditions, the difference between the two resting states remained relatively small. While some fMRI conditions, such as the two resting states, provided relatively stable prediction accuracy across all included outcomes, others, such as EN‐back, showed stronger variability in their predictability for different neuropsychological outcomes. This suggested that task‐based fMRI conditions may introduce greater differences in predictive performance depending on the neuropsychological measure under consideration.

**FIGURE 5:**
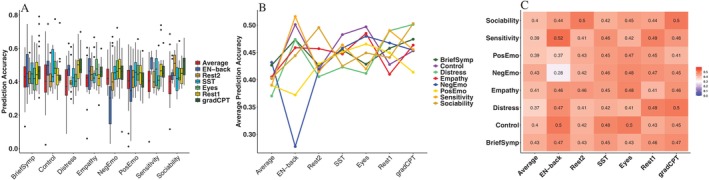
Prediction accuracy across tasks and categories. (A) Boxplots show prediction accuracy distributions for different tasks within each category. Colors represent various tasks, illustrating differences in variability and central tendencies. (B) Line plot depicts average prediction accuracy by category across tasks, with each line representing a category. This highlights performance trends and variations among categories over tasks. (C) Each cell represents the mean accuracy when predicting a particular behavioral category (*y* label) using a particular fMRI condition (*x* label). Calculation of mean prediction accuracy utilizes the 5 replications of all behavioral variables in each behavioral category.

We further illustrated the particular pairing of fMRI conditions and neuropsychological measures in Figure 5. In Figure 5C, we reported the average prediction accuracy across 5 replications when predicting a particular neuropsychological category (*y* label) using a particular fMRI condition (*x* label). We observed that certain tasks were more relevant to some neuropsychological categories and should be prioritized when predicting outcomes in these contexts. When predicting outcomes using the EN‐back fMRI data, the NegEmo category exhibited substantially lower accuracy than others. The EN‐back condition achieved nearly 0.5 prediction accuracy for the Sensitivity category while only 0.27 for the NegEmo category. A further investigation of indicators of the NegEmo category revealed the average prediction accuracy for fatigue, fear, guilt, hostility, negative affect, sadness, and shyness was 0.35, 0.12, 0.11, 0.18, 0.39, 0.50, and 0.33, respectively. The average prediction accuracy was calculated as the mean of five‐fold cross‐validation. We noticed that EN‐back provided high predictions for negative effects, but low prediction accuracy for negative feelings such as fear, guilt, and hostility. The lower prediction accuracy for these negative emotions indicated that the connectome under the EN‐back task condition contained limited negative feelings‐related features, making EN‐back less optimal for investigating negative emotions.

To answer the question—which fMRI condition, resting and task included, could best predict a specific category of neuropsychological measures—we coded the fMRI task conditions as predictors of a regression model explaining differences in prediction accuracies (averaged across 5 replications) associated with each category of neuropsychological measures. We reported the results in Table S2. With Rest 1 as the reference condition, a larger coefficient indicated a comparatively higher averaged prediction accuracy under the corresponding fMRI condition. With different fMRI conditions as predictors, the NegEmo category had the largest $R^{2}$, which suggested there was substantial variation in the predictability of different fMRI conditions for NegEmo measures. We found a large negative coefficient, −0.190, for the EN‐back condition, suggesting that EN‐back showed substantially lower prediction accuracy in predicting NegEmo than Rest 1. This result corresponded with the result shown in Figure 5. Together, our results suggested that similarities and differences existed in the predictability of fMRI task and resting conditions.

### 3.2 Neuropsychological Measures Show Shared and Distinct Functional Fingerprints

**FIGURE 6:**
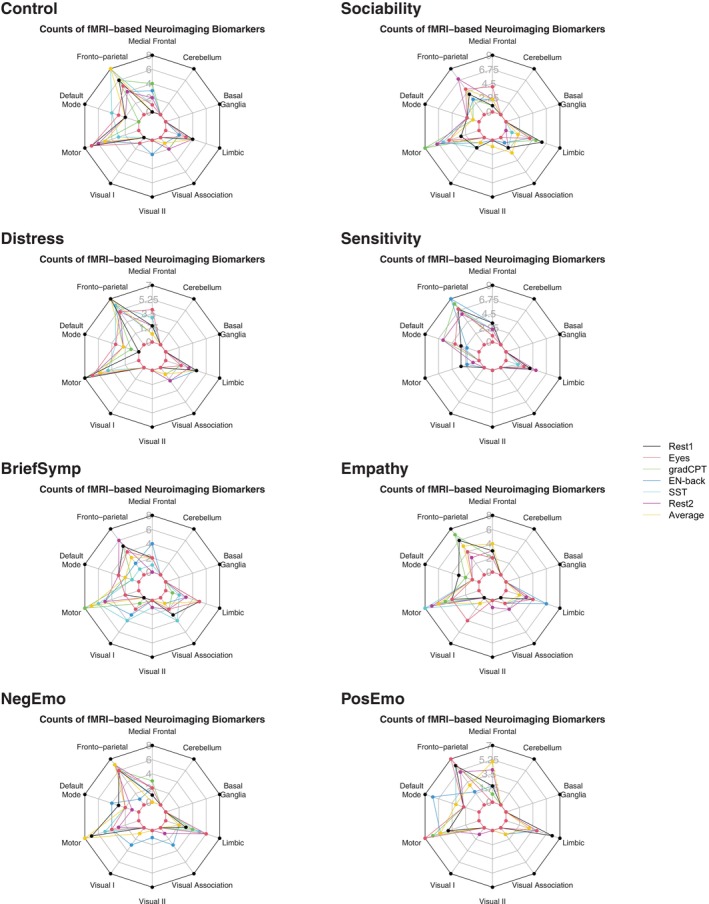
Spidar plot of functional biomarkers for behavioral categories. This figure displays counts of functional biomarkers across different behavioral categories: Control, Sociability, Distress, Sensitivity, BriefSymp, Empathy, NegEmo, and PosEmo. Each plot represents the distribution of various functional biomarkers in specific anatomical regions, with different colored lines indicating different fMRI conditions. The number of functional biomarkers is derived from the functional labels assigned to the top 20 brain nodes. The top 20 brain nodes refer to the top 10 brain nodes with the largest positive covariance and the top 10 brain nodes with the smallest negative covariance. The covariance estimates between 268 brain nodes and behavior categories are calculated by the latentSNA model.

In Figure 6, we presented spider plots for the number of top 20 functional biomarkers in ten functional systems for each category of neuropsychological measure including Control, Sociability, Distress, Sensitivity, BriefSymp, Empathy, NegEmo, and PosEmo. The corresponding bar plots were provided in Figure S2. We used these plots to summarize similarities and differences in patterns of biomarkers across functional systems among neuropsychological categories. Figure 6 showed that there are both consistent patterns and notable differences in functional biomarkers across neuropsychological categories. In the following, we provided a thorough examination of these patterns to gain insights into the neural underpinnings of various neuropsychological outcomes and identified potential biomarkers for specific neuropsychological categories.

In Figure 6, we consistently identified fronto‐parietal, motor, and limbic functional systems to be associated with neuropsychological measures. The fronto‐parietal network was found to contribute to individual differences for a range of neuropsychological categories using most fMRI conditions. Another significant functional network identified was the motor system, which was found to be associated with emotion‐related behaviors. Specifically, the number of brain regions in the motor functional system was comparable to that of the fronto‐parietal system associated with the NegEmo, PosEmo, and Distress categories, suggesting that both positive and negative emotions were associated with brain regions in the motor functional system. The limbic system was also found to be associated with neuropsychological measures. The limbic system was associated with NegEmo, PosEmo, Empathy, and Sociability categories, replicating the previous literature supporting the role of the limbic system in emotions (Rolls 2015).

Despite the similarities in the functional fingerprints associated with neuropsychological measures, there were differences as well. It was interesting that the motor functional system was consistently absent in explaining individual differences in Sensitivity across independent replications of the same model and across replications with different fMRI conditions, which contrasted sharply with its role in other neuropsychological categories. The Sensitivity category encompassed both neutral perceptual sensitivity and affective perceptual sensitivity, each tied to perceptual processes. Additionally, when comparing sensitivity with other neuropsychological measures, the default mode network was found to be dominantly involved in neuropsychological outcomes, especially in the resting state.

To provide secondary support for the identified shared and distinct functional fingerprints associated with neuropsychological measures, we conducted a robustness check using replication level data—which compared with cross‐validated results, shows the model replicability with random sampling of the training and test data. The regression results revealed significant associations that reinforced our previous observations (see details in Table S5 of the supporting information). From Table S5, we found that brain nodes in the fronto‐parietal and motor networks exhibited stronger associations with a range of outcomes. This conclusion was drawn from regressing the absolute values of brain node covariance on network dummy variables, where the coefficients for fronto‐parietal and motor networks showed larger magnitudes. These results further reinforced our previous findings that the fronto‐parietal and motor networks are involved across categories. To provide supplementary analyses, we examined the functional biomarker effects on covariance magnitude across all behaviors (irrespective of behavioral category differences) in Table S4, and investigated how different fMRI conditions modulate the covariance between brain activity and behavioral measures in Table S6.

### 3.3 Resting and Task fMRI Conditions Reveal Shared and Distinct Brain‐Behavior Links

**FIGURE 7:**
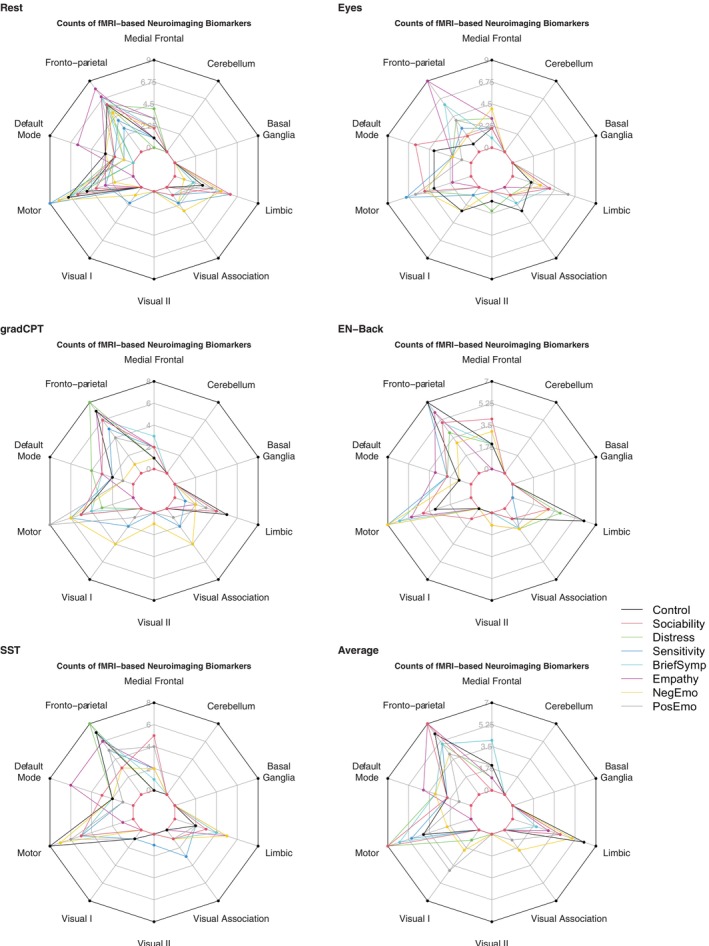
Spider plot of functional biomarkers for each fMRI condition. This figure displays counts of functional biomarkers of top 20 brain nodes (The definition of top 20 brain nodes is consistent with that specified in Figure 6) across different fMRI conditions: Rest, Eyes, gradCPT, EN‐back, SST, and Average. Each plot represents the distribution of various functional biomarkers, with different colored lines indicating different behavioral categories, including Control, Sociability, Distress, Sensitivity, BriefSymp, Empathy, NegEmo, and PosEmo.

Having compared the different brain links among behavioral categories, we then examined differences among specific fMRI conditions. The difference among fMRI conditions was measured by the counts of functional biomarkers among the top 20 brain nodes. The top 20 brain nodes were identified as the combination of the top 10 brain nodes with the highest positive covariance and the top 10 brain nodes with the lowest negative covariance. We first calculated the number of biomarkers for each neuropsychological measure under each fMRI condition using five‐fold cross‐validation. Then, we averaged the number of biomarkers by fMRI conditions and reported results in Table S7. Figure 7 visualized the counts of functional biomarkers reported in Table S7. It showed that the Eyes fMRI condition displayed a different brain‐behavior link pattern than other task conditions. While gradCPT, EN‐back, and SST showed predominantly fronto‐parietal and motor biomarkers, the Eyes condition additionally displayed default mode and medial frontal involvement. The average condition appeared to occupy an intermediate position between rest and task conditions. More specifically, the Average condition had a similar number of fronto‐parietal functional biomarkers as the Rest condition. However, in terms of visual biomarkers, it was closer to that of the gradCPT and Eyes task‐based conditions than the Resting State.

We further investigated the covariance estimates for each of the 268 brain regions, representing the strength of their associations with the Sociability category in Figure 3. We focused on Sociability for further investigation because, on average, the Sociability category resulted in the highest absolute value of covariance estimates of brain nodes under all fMRI conditions. Besides the Sociability measure, we also provided a comprehensive analysis with additional neuropsychological measures, including Control and Sensitivity, which can be found in Figures S4 and S5. The circle plot (see Figure 4) showed the top 5 positive and top 5 negative functional connectivity edges associated with Sociability. We also did a corresponding brain plot for each circle plot, showing the position of the top 20 brain nodes and their covariance estimates with the Sociability category.

Brain connectome under the Eyes condition showed the strongest associations with Sociability. In Figure 3, a lighter color suggested that this brain region had stronger links with Sociability measures. The light yellow and light blue meant the brain region was positively or negatively associated with behavior variables within the Sociability category correspondingly. Across fMRI conditions, we found the rest condition showed generally lower association with Sociability than task conditions. This observation suggested that task‐based connectomes contained more information about Sociability. The brain plot for the Average condition showed that its association with Sociability fell between those of the rest and task conditions. Among the four task conditions, the Eyes condition had the strongest link with Sociability.

To answer this question of whether the Eyes condition consistently showed strong associations with a range of neuropsychological measures, we analyzed Control (Figure S4) and Sensitivity (Figure S5). Control was selected for its focus on cognitive behaviors, often contrasted with the Sociability dimension. Figure S3 showed that the Eyes condition had the highest covariance estimates. The SST condition, with higher covariance estimates for Control than Sociability, remained lower than the Eyes condition. We examined Sensitivity due to its unique biomarker profile in Figure 6. Figure S4 revealed that strong links exist between the brain and Sensitivity under both Eyes and gradCPT conditions. These results confirmed that brain connectivity had the strongest associations with multiple neuropsychological measures under the Eyes condition, including Sociability, Sensitivity, and Control. GradCPT showed a notable association with Sociability and Sensitivity, but a weak association with Control. Together, our results show that certain tasks may be more optimal for specific outcomes than others.

Figure 3 and Figure S3 compared differences in the functional biomarkers across fMRI conditions. Figure S3 used different colors to classify different functional regions in brain plots. By matching the location of lighter brain regions in Figure 3 with the functional regions in Figure S3, we noticed that under many fMRI conditions, a large proportion of brain regions with lighter colors (indicating stronger association with Sociability) were located in fronto‐parietal areas. This observation aligned with Figure 7, demonstrating the significance of fronto‐parietal biomarkers for the Sociability category. Despite this general pattern, different fMRI conditions exhibited unique features. The Eyes condition had high connectivity nodes in the fronto‐parietal, motor, and limbic regions. For gradCPT, EN‐back, and SST, high covariance nodes were mainly in fronto‐parietal areas. The Rest condition showed high covariance in default mode and fronto‐parietal regions, while the Average condition had large covariance nodes across default mode, fronto‐parietal, and motor regions.

Figure 4 revealed distinct patterns of brain connectivity associated with Sociability across various fMRI conditions, highlighting the complex and task‐dependent nature of social cognition in the brain. This figure displayed connectivity among top Sociability‐associated brain nodes. Red lines showed connections between top 5 positive nodes; blue lines for top 5 negative nodes. For the rest condition, we observed positive connections (red) in frontal and temporal regions, while negative connections (blue) in occipital and cerebellar areas. GradCPT exhibited strong frontal and temporal connectivity, consistent with its role in attention and cognitive control. EN‐back displayed widespread connectivity across multiple brain regions, reflecting the complex cognitive processes involved in this working memory task. The Eyes condition showed distinct connectivity patterns, with strong positive connections among prefrontal, limbic, and motor regions. This unique pattern might be related to the social and emotional processing demands of this task. SST demonstrated notable prefrontal‐cerebellar positive connectivity and parietal–temporal negative connectivity, possibly reflecting the inhibitory control processes central to this task. In addition to conducting detailed comparisons among specific fMRI conditions, we provided a broader analysis comparing resting state and task‐based fMRI conditions by utilizing the averaged values from two resting state conditions and four task conditions. The results are summarized in Figure S6.

## 4 Discussion

Many pieces of evidence suggest that predictive models built using task‐based fMRI data generally outperform those relying on resting‐state fMRI. This superiority can be attributed to the ability of task‐based scans to directly capture brain activity associated with specific cognitive functions, while resting‐state fMRI relies on spontaneous neural fluctuations that may not always be directly linked to the cognitive or behavioral traits of interest (Sripada et al. 2020). Task‐based paradigms are specifically designed to engage neural circuits responsible for cognitive processing, thereby allowing researchers to extract functionally relevant signals that are more informative for predictive modeling (Greene et al. 2018). In contrast, resting‐state fMRI primarily captures intrinsic functional connectivity, which, while useful for understanding the brain's overall network architecture, may not always provide task‐relevant information necessary for high‐precision predictions of cognitive performance (Gal, Tik, et al. 2022).

A key advantage of task‐based fMRI lies in its ability to elicit robust and consistent neural responses across individuals, reducing inter‐subject variability and increasing the generalizability of predictive models (Zhao et al. 2023). The evidence suggests that neural activation patterns recorded during cognitively demanding tasks, such as the N‐back working memory task, exhibit strong correlations with general cognitive ability, outperforming connectivity patterns derived from resting‐state scans (Sripada et al. 2020). This finding underscores the notion that externally driven neural states may serve as a more reliable basis for characterizing cognitive function compared to spontaneous fluctuations that occur in the absence of explicit task engagement (Gal, Coldham, et al. 2022).

Moreover, task‐induced brain states have been shown to enhance the predictive power of individual traits, suggesting that cognitive tasks amplify meaningful neural differences between individuals that might otherwise be obscured in resting‐state conditions (Greene et al. 2018). The structured nature of task‐based fMRI allows for a more direct mapping between neural activity and cognitive processes, facilitating stronger associations between brain function and behavior. Recent work comparing naturalistic stimuli‐driven fMRI (e.g., movie‐watching) with resting‐state scans further reinforces this perspective, as functional connectivity derived from task‐based conditions was found to be superior in predicting individual differences in brain activity (Gal, Coldham, et al. 2022). This highlights the potential of using externally controlled paradigms to optimize predictive models, particularly in studies focused on cognitive and clinical outcomes.

However, it is also important to acknowledge the limitations and challenges of task‐based fMRI. While task paradigms provide clearer functional interpretations, they are constrained by the specific cognitive functions they target, making them less flexible compared to resting‐state scans, which can be used to investigate a wide range of cognitive and clinical traits without requiring a tailored task design (Gal, Tik, et al. 2022). Additionally, task‐based fMRI can be influenced by subject‐specific variability in task performance, motivation, and compliance, introducing potential confounds that may affect predictive accuracy (Zhao et al. 2023). Despite these challenges, the empirical evidence strongly suggests that, when designed appropriately, task‐based fMRI provides richer and more targeted information for prediction compared to resting‐state fMRI, ultimately leading to improved modeling of cognitive and behavioral outcomes (Sripada et al. 2020).

In this study, we aimed to address whether and how we could improve the cost‐effectiveness of fMRI‐based neuroimaging studies by using tailored fMRI tasks during data collection to investigate a range of neuropsychological measures. With generally satisfactory predictive power obtained by fitting our Bayesian generative network model for connectome‐based predictive modeling, we had a robust foundation to investigate differences in predictive power and associated neuroimaging biomarkers between resting and various task conditions. Notably, we found a lower prediction accuracy averaging across fMRI conditions than across other fMRI conditions. This reduction in accuracy may have been attributed to the averaging effect, which minimized the unique brain circuit perturbations from well‐designed fMRI tasks related to different neuropsychological categories. In addition, we found limited prediction accuracy using the EN‐back condition to model certain negative emotions. This finding highlighted that specific fMRI conditions may be more appropriate for predicting certain neuropsychological categories than others; there was a unique pairing of fMRI tasks and neuropsychological measures that should not be ignored when designing well‐powered fMRI studies. We compared optimal fMRI conditions for each of the eight neuropsychological categories included in the study: BriefSymp, Control, Distress, Empathy, NegEmo, PosEmo, Sensitivity, and Sociability. This insight was essential for other researchers aiming to achieve higher power when studying specific outcomes with limited resources.

When comparing functional fingerprints associated with different neuropsychological outcomes, our study revealed that neuropsychological categories shared common functional biomarkers but maintained distinct differences. For instance, the fronto‐parietal and motor functional systems were found to be associated with a range of neuropsychological outcomes with exceptions. The fronto‐parietal network demonstrated consistent involvement across multiple cognitive and emotional domains, aligning with its established role in attention control (Wang et al. 2010) and executive function processing (Hearne et al. 2016; Naghavi and Nyberg 2005). Similarly, the motor system showed robust associations with both positive and negative emotional processing (Melzer et al. 2019; Morecraft et al. 2004), suggesting its broader involvement in affective regulation beyond traditional motor control functions (Blakemore and Vuilleumier 2017). The motor functional system was not found to contribute to sensitivity outcomes, a finding that contrasted with developmental literature linking motor ability and perceptual development (Bushnell and Boudreau 1993). These unique biomarker profiles offered insights into the distinct characteristics of each neuropsychological category, particularly in the context of network‐specific contributions to behavioral outcomes (Raichle 2015), suggesting avenues for future research. The identification of these category‐specific neural signatures, characterized by differential engagement of major functional systems (Rolls 2015), could help identify objective diagnostic features for psychiatric disorders.

In addition to comparing neuropsychological categories, we also conducted a comparable analysis of the neuroimaging biomarkers under different fMRI conditions. We found that the Eyes condition had the most medial‐frontal biomarkers across task conditions. EN‐back showed smaller involvement in the default mode network than others. Interestingly, we observed that the Eyes condition showed strong brain‐behavior links with a range of behaviors including control, sensitivity, and sociality. GradCPT demonstrated strong links with sensitivity and sociability but weaker links with control. The Eyes condition was found with strong predictive power for a range of outcomes.

Our study had limitations. The classification of behavioral categories may not have been sufficiently accurate, and there may have been inherent differences among variables in their psychometric properties within each neuropsychological category. Future studies should focus on refining behavioral classifications and exploring within‐category variations to enhance the precision of predictions and biomarker identification. Fitting all neuropsychological measures together in one model is a another direction for our future work, which could provide a more comprehensive understanding of the complex interactions between different psychological constructs. Another limitation of our study is the use of Pearson correlation coefficients to construct functional brain networks, which may be influenced by potential confounders that artificially inflate functional connectivity estimates. Factors such as head motion, physiological noise (e.g., cardiac and respiratory fluctuations), and global signal variations could contribute to spurious correlations between brain regions. Although we employed standard preprocessing steps, including motion correction and nuisance signal regression, these approaches may not entirely eliminate the effects of such confounders. Future studies could benefit from alternative functional connectivity metrics, such as partial correlation or multivariate approaches, that explicitly account for confounding factors. Additionally, leveraging more sophisticated motion denoising techniques or independent component analysis may help reduce the risk of inflated connectivity and improve the robustness of network‐based analyses. Finally, regarding the selection of the training sample, we acknowledge that the diverse psychiatric conditions of participants—ranging from healthy individuals to those with multiple comorbid disorders—may significantly impact the model's results. In our study, the random split between the training and testing samples may overlook potential influences of specific psychiatric conditions, which could affect the generalizability and interpretability of the findings. This represents a limitation of our approach, as a more stratified sampling strategy might better account for these variations.

## Supporting information


Data S1.

## Data Availability

The data that support the findings of this study are available on request from the corresponding author. The data are not publicly available due to privacy or ethical restrictions.
