# Supplementary material for: Cost Efficiency of fMRI Studies Using Resting‐State Vs. Task‐Based Functional Connectivity
Source: Hum Brain Mapp. 2025 Jun 21;46(9):e70260. doi: 10.1002/hbm.70260 (PMC12182254; doi:10.1002/hbm.70260)
Supplement: Supplementary file 1 — Data S1. [file HBM-46-e70260-s001.pdf]

Supplementary Materials

March 13, 2025

1 Additional Information

1.1 Identifiability Considerations and Estimation Strategy

We address the identifiability challenge concerning the latent variables $\boldsymbol{\gamma_j}$ in the data generation process for connectivity matrices C_j, where $\gamma_{x,j}$ and $\gamma_{y,j}$ represent positions for brain regions with indices $x, y = 1, 2, ..., 268$. This challenge stems from the bilinear interaction structure, which introduces a reflection indeterminacy. Mathematically, for any estimated latent variables $\hat{\gamma}_{x,j}$ and $\hat{\gamma}_{y,j}$, their negations $-\hat{\gamma}_{x,j}$ and $-\hat{\gamma}_{y,j}$ yield identical products and therefore equivalent likelihood values:

$$(\hat{\gamma}_{x,j})(\hat{\gamma}_{y,j}) = (-\hat{\gamma}_{x,j})(-\hat{\gamma}_{y,j}) \tag{1}$$

This sign ambiguity manifests during MCMC estimation, where the signs of $\gamma_{x,j}$ can flip while preserving the same connectivity values. The ambiguity particularly affects our ability to consistently estimate the correlation between the latent brain position variables $\gamma_{x,j}$ and the behavioral latent variable κ_j.

To resolve this identifiability issue, we implement a sequential estimation approach:

1. First, we allow a sufficient burn-in period during which the MCMC chains explore the parameter space and the signs of $\gamma_{x,j}$ converge toward optimal values.

2. Subsequently, we fix the signs of all $\gamma_{x,j}$ to match those from the first iteration following the burn-in period.

This two-stage strategy effectively stabilizes the estimation process. The latent variables become "softly identified" through their relationship with behavioral outcomes, as the signs of $\gamma_{x,j}$ must maintain consistent correlation patterns with κ_j. This relationship provides a natural anchor for identification after the burn-in period has been completed.

1.2 Additional Description for SST fMRI condition

The Stop-Signal Task (SST) is a widely used task for assessing response inhibition. Originally introduced as an experiment task to study visual reaction time, SST was later formalized within the independent race model that conceptualizes response inhibition as a race between a go process and a stop process, and a successful inhibition occurs when the stop process finishes first (Lappin & Eriksen, 1966; Logan & Cowan, 1984). In a typical SST, participants perform a primary "go" task—pressing a key in response to an arrow pointing left or right—while being occasionally presented with a stop signal, such as an auditory tone or a visual cue, after a variable stop-signal delay that instructs them to inhibit their response. The Stop-Signal Reaction Time, a measure of the latency of the stopping process, is the key dependent variable used to assess inhibitory control (Logan & Cowan, 1984).

SST has been widely implemented to measure response inhibition (Verbruggen & Logan, 2019). In particular, SST has been widely applied in the study of inhibitory control deficits across various neuropsychiatric conditions, including attention-deficit/hyperactivity disorder (ADHD), obsessive-compulsive disorder (OCD), and substance use disorders (Bari & Robbins, 2013; Lipszyc & Schachar, 2010). SST performance differential is associated with difference in impulsivity and cognitive control, making SST a valuable tool for investigating behavioral regulation. Neuroimaging studies have linked SST to key brain regions involved in response inhibition, including the right inferior frontal gyrus, pre-supplementary motor area, and basal ganglia (Aron, 2011; Chambers et al., 2009). These findings highlight the importance of SST in understanding the neural mechanisms underlying impulse control and its dysfunction in clinical populations (Verbruggen, 2019).

2 Additional Figures

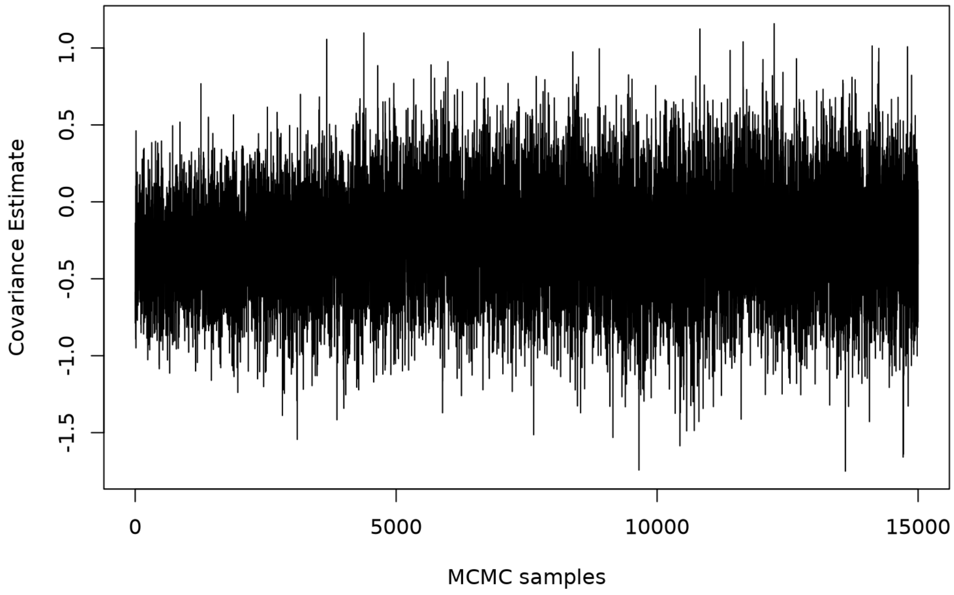

Figure S1: **Traceplot: covariance estimate between anxiety and a brain node under gradCPT task** The x-axis represents 15000 MCMC samples, and the y-axis quantifies the covariance estimate between anxiety variable and a brain node under gradCPT task.

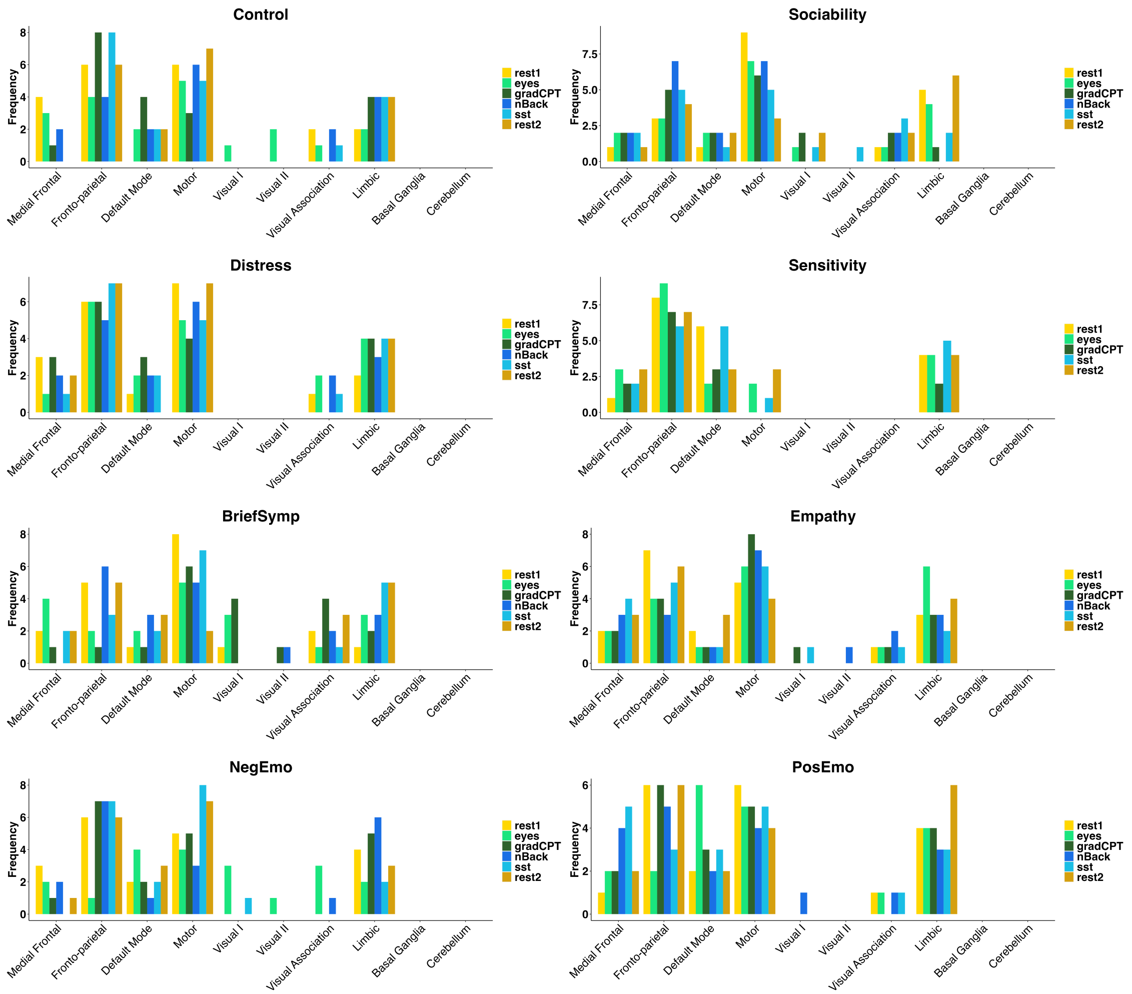

Figure S2: **Barplot of Functional Biomarkers by Outcome Category** These bar plots present the distribution of functional biomarkers across behavioral categories, with distinct colors representing different fMRI conditions within each category-specific plot. The visualization demonstrates our analytical pipeline: for each behavioral category, we derive predictions using functional connectivity matrices obtained under distinct fMRI conditions. For each model fitting, we identify the top 20 brain regions exhibiting maximal absolute covariance with behavioral measures, then aggregate their functional network assignments to generate condition-specific biomarker profiles.

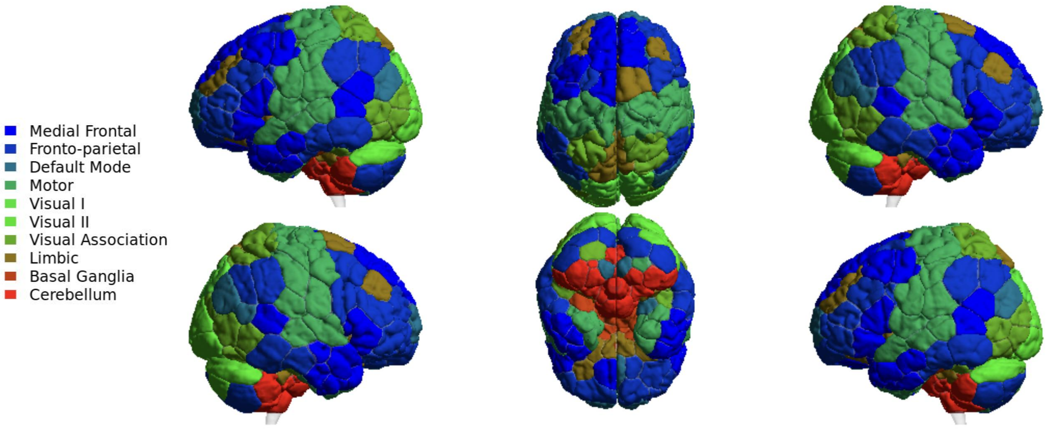

Figure S3: **Brain plot for Anatomical Regions** This visualization depicts distinct functional networks overlaid on a standardized brain template. Different colors delineate specific functional systems: Medial Frontal (dark blue), Fronto-parietal (blue), Default Mode (teal), Motor (green), Visual I/II (light green), Visual Association (olive), Limbic (brown), Basal Ganglia (red), and Cerebellum (dark red). Multiple views illustrate the spatial distribution of these functional networks across different anatomical perspectives.

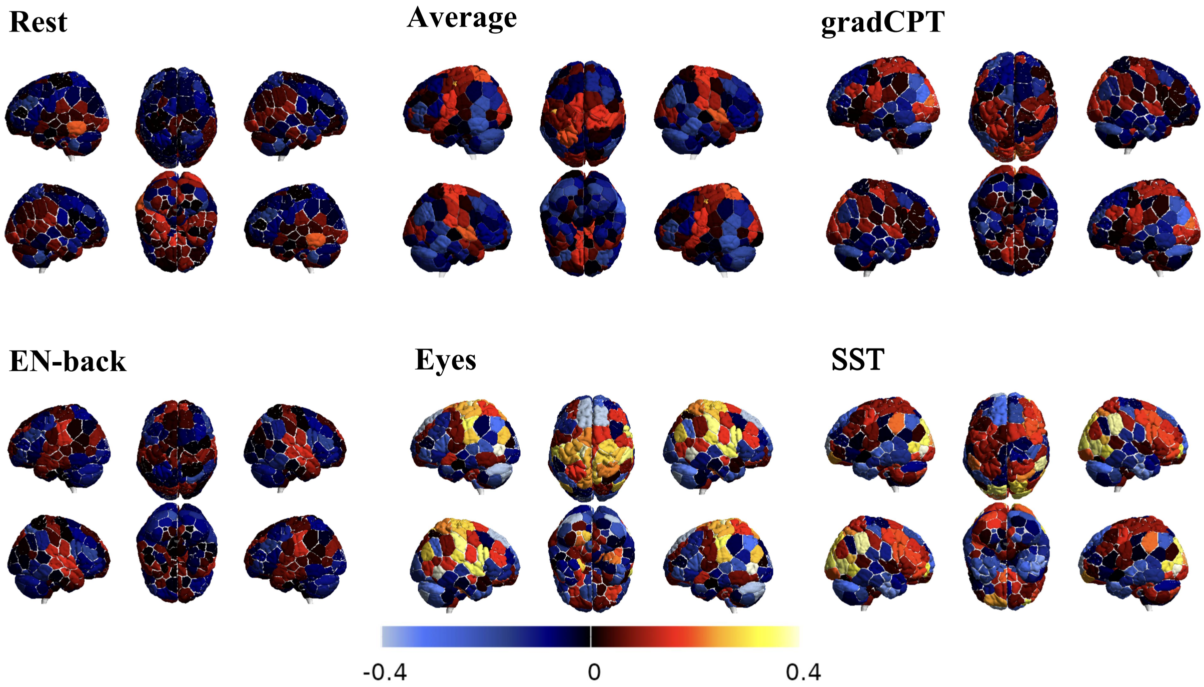

Figure S4: **Covariance Estimates Between Brain Regions and Control Across Various fMRI Conditions** This figure visualizes the covariance estimates of 268 brain nodes across different fMRI conditions: Rest, Average, gradCPT, EN-back, Eyes, and SST. The color scale indicates the magnitude of covariance, ranging from -0.4 (blue) to 0.4 (yellow). A lighter color indicates higher absolute value of covariance.

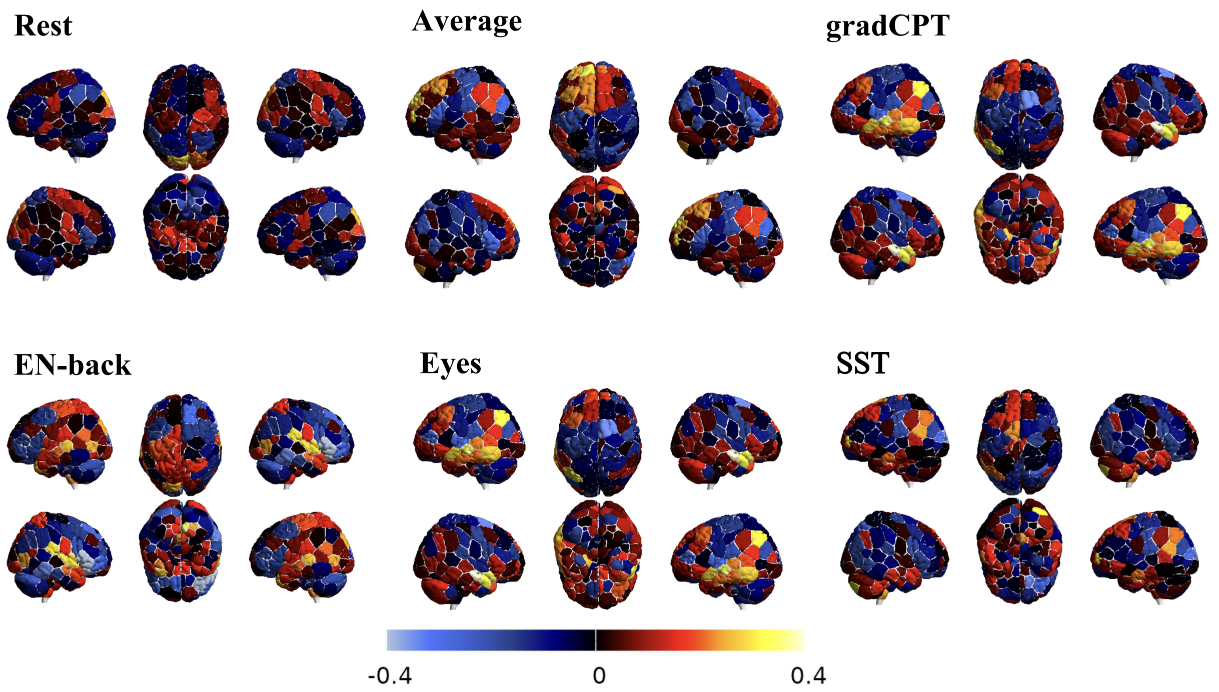

Figure S5: **Covariance Estimates Between Brain Regions and Sensitivity Across Various fMRI Conditions** This figure visualizes the covariance estimates of 268 brain nodes across different fMRI conditions: Rest, Average, gradCPT, EN-back, Eyes, and SST. The color scale indicates the magnitude of covariance, ranging from -0.4 (blue) to 0.4 (yellow). A lighter color indicates higher absolute value of covariance.

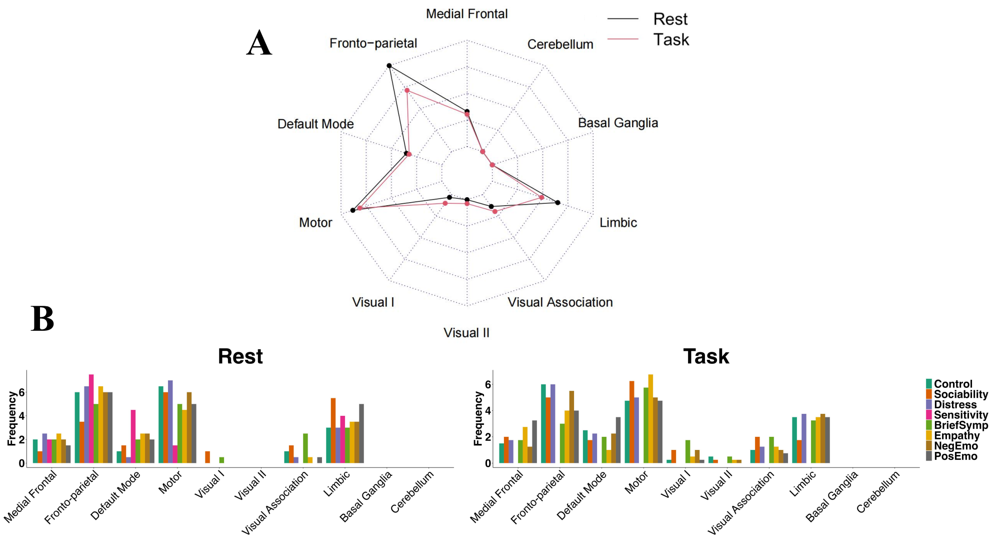

Figure S6: **Comparison of Functional Biomarkers by Rest and Task Condition** Panel A shows a spider plot comparing the distribution of functional biomarkers between Rest (black line) and Task (red line) conditions across various anatomical regions. Panel B presents bar plots of the frequency of different functional biomarkers in the Rest and Task conditions, with each color representing a different behavioral category (Control, Sociability, Distress, Sensitivity, BriefSymp, Empathy, NegEmo, PosEmo). For frequency quantification, the methodology involves counting the functional labels assigned to the top 20 brain nodes under each fMRI condition. The procedure then implements averaging of these counts across all task conditions and both resting states to derive the final frequencies for Rest and Task.

3 Additional Tables

This section can include tables that are supplementary to the main text.

**Diagnosis**	**Number of Patients**
Depression	36
Anxiety disorders	21
Attention Deficit Hyperactivity Disorder (ADHD)	16
Bipolar Disorder	14
Schizophrenia	10
Borderline personality disorder	6
Panic disorder	5
Post-Traumatic Stress Disorder (PTSD)	3
Obsessive-Compulsive Disorder (OCD)	1
Antisocial personality disorder	1
Avoidant personality disorder	1
Anorexia nervosa	1

Table S1: **Summary of Patient Diagnoses** The table presents frequency counts for each diagnosed disorder, with depression (n=36) and anxiety disorders (n=21) being the most prevalent conditions. Some patients are diagnosed with multiple disorders, resulting in diagnosis counts exceeding the total number of patients.

Variable	BriefSymp	NegEmo	PosEmo	Empathy	Distress	Sociability	Control	Sensitivity
Intercept	0.426^{***}	0.467^{***}	0.449^{***}	0.410^{***}	0.490^{***}	0.440^{***}	0.428^{***}	0.490^{***}
	(0.021)	(0.026)	(0.027)	(0.021)	(0.034)	(0.028)	(0.036)	(0.033)
Rest2	−0.031	−0.050	−0.020	0.047	$-0.084^{\cdot}$	0.055	−0.008	−0.075
	(0.029)	(0.037)	(0.039)	(0.030)	(0.048)	(0.040)	(0.051)	(0.047)
Average	−0.031	−0.035	−0.043	0.011	-0.112^{*}	−0.038	−0.025	$-0.100^{\cdot}$
	(0.029)	(0.037)	(0.039)	(0.030)	(0.048)	(0.040)	(0.051)	(0.047)
EN-back	0.016	-0.190^{***}	$-0.077^{\cdot}$	0.049	−0.016	0.003	0.073	0.026
	(0.029)	(0.037)	(0.039)	(0.030)	(0.048)	(0.040)	(0.051)	(0.047)
SST	−0.003	−0.010	0.002	0.037	−0.067	−0.016	0.055	−0.026
	(0.029)	(0.037)	(0.039)	(0.030)	(0.048)	(0.040)	(0.051)	(0.047)
Eyes	−0.029	0.012	0.016	0.075^{*}	−0.078	0.008	0.069	−0.070
	(0.029)	(0.037)	(0.039)	(0.030)	(0.048)	(0.040)	(0.051)	(0.047)
gradCPT	0.017	−0.013	−0.036	$0.053^{\cdot}$	0.012	0.062	0.025	−0.035
	(0.029)	(0.037)	(0.039)	(0.030)	(0.048)	(0.040)	(0.051)	(0.047)
**R-squared**	0.106	0.493^{***}	0.192	0.297	0.362	0.408	0.340	0.433

Standard errors in parentheses

Signif. codes: 0 '***' 0.001 '**' 0.01 '*' 0.05 '.' 0.1 ' ' 1

Note. BriefSymp: Psychological Symptoms Inventory, NegEmo: Negative Emotional Spectrum, PosEmo: Positive Emotional Spectrum, Empathy: Empathy Engagement Scale, Distress: Emotional Distress Spectrum, Sociability: Sociability and Vitality Scale, Control: Self-Regulation Control Measures, Sensitivity: Sensory and Emotional Awareness Scale.

Table S2: **Regression of Prediction Accuracy on Task Labels for Each Category (Reference: Rest1)** Regression coefficients and standard errors from linear models predicting behavioral category-specific prediction accuracies using different fMRI conditions, with Rest1 as the reference condition. Each column represents a separate regression model for each behavioral category. The intercept represents the baseline prediction accuracy under Rest1 condition, while other coefficients indicate changes in prediction accuracy relative to Rest1.

Variable	BriefSymp	NegEmo	PosEmo	Empathy	Distress	Sociability	Control	Sensitivity
Intercept	0.426***	0.418***	0.429***	0.457***	0.406***	0.496***	0.420***	0.415***
	(0.014)	(0.016)	(0.019)	(0.019)	(0.020)	(0.023)	(0.027)	(0.023)
Rest1	0.031	0.050*	0.020	−0.047	0.084**	−0.055$^{\cdot}$	0.008	0.075*
	(0.020)	(0.023)	(0.026)	(0.027)	(0.028)	(0.032)	(0.038)	(0.032)
Average	0.000	0.014	−0.039	−0.052$^{\cdot}$	−0.035	−0.092**	−0.017	−0.025
	(0.020)	(0.023)	(0.028)	(0.028)	(0.028)	(0.032)	(0.038)	(0.032)
EN-back	0.047*	−0.140***	−0.057*	0.002	0.068*	−0.051	0.081*	0.101**
	(0.020)	(0.023)	(0.026)	(0.027)	(0.028)	(0.032)	(0.038)	(0.032)
SST	0.028	0.040$^{\cdot}$	0.022	−0.010	0.017	−0.071*	0.063$^{\cdot}$	0.049
	(0.020)	(0.023)	(0.026)	(0.027)	(0.028)	(0.032)	(0.038)	(0.032)
Eyes	0.002	0.062**	0.036	0.028	0.006	−0.046	0.077*	0.005
	(0.020)	(0.023)	(0.026)	(0.027)	(0.028)	(0.032)	(0.038)	(0.032)
gradCPT	0.048*	0.037	−0.015	0.006	0.096***	0.007	0.033	0.040
	(0.020)	(0.023)	(0.026)	(0.027)	(0.028)	(0.032)	(0.038)	(0.032)
**R-squared**	0.044*	0.306***	0.092**	0.093*	0.218***	0.135*	0.119*	0.194**

Standard errors in parentheses

Signif. codes: 0 '***' 0.001 '**' 0.01 '*' 0.05 '.' 0.1 ' ' 1

Note. BriefSymp: Psychological Symptoms Inventory, NegEmo: Negative Emotional Spectrum, PosEmo: Positive Emotional Spectrum, Empathy: Empathy Engagement Scale, Distress: Emotional Distress Spectrum, Sociability: Sociability and Vitality Scale, Control: Self-Regulation Control Measures, Sensitivity: Sensory and Emotional Awareness Scale.

Table S3: **Regression of Prediction Accuracy on Task Labels for Each Category (Reference: Rest2)** Parallel regression analysis using Rest2 as the reference condition to validate the robustness of task-specific effects. Model specifications and interpretations are identical to Table S4, with coefficients representing changes in prediction accuracy relative to Rest2 baseline.

Coefficient	Estimate	Std. Error	t value	Pr(> $\|t\|$)
(Intercept)	-0.030871	0.003127	-9.872	$< 2e-16$ ***
Medial Frontal	-0.005684	0.004065	-1.398	0.162
Fronto-parietal	0.065978	0.003941	16.741	$< 2e-16$ ***
Motor	0.029553	0.003700	7.987	$1.40e-15$ ***
Visual I	0.005119	0.004544	1.127	0.260
Visual II	-0.023928	0.005613	-4.263	$2.02e-05$ ***
Visual Association	0.035722	0.004544	7.862	$3.84e-15$ ***
Limbic	0.067634	0.004037	16.753	$< 2e-16$ ***
Basal Ganglia	0.017629	0.004065	4.337	$1.45e-05$ ***
Cerebellum	0.051504	0.004011	12.841	$< 2e-16$ ***

Table S4: **Regression Analysis of Functional Biomarker Effects on Covariance Magnitude** Regression coefficients quantify the association between functional network assignments and the absolute magnitude of covariance estimates. Positive coefficients indicate that brain regions assigned to the corresponding functional network exhibit systematically larger absolute covariance values, suggesting stronger network-specific contributions to brain-behavior relationships.

Variable	BriefSymp	NegEmo	PosEmo	Empathy	Distress	Sociability	Control	Sensitivity
Intercept	-0.007	−0.045***	−0.061***	-0.015	−0.033***	0.022.	−0.078	−0.034*
	(0.004)	(0.007)	(0.007)	(0.011)	(0.010)	(0.012)	(0.014)	(0.014)
Medial Frontal	0.011.	0.007	0.005	−0.058***	0.005	−0.013	−0.013	−0.037*
	(0.006)	(0.009)	(0.009)	(0.014)	(0.013)	(0.016)	(0.018)	(0.018)
Fronto-parietal	0.049***	0.086***	0.072***	0.086***	0.070	0.015	0.089***	0.057
	(0.006)	(0.009)	(0.009)	(0.014)	(0.012)	(0.015)	(0.018)	(0.017)
Motor	−0.038***	0.014***	0.157***	−0.023.	0.006	−0.005	0.123***	0.063***
	(0.006)	(0.009)	(0.009)	(0.013)	(0.012)	(0.014)	(0.016)	(0.016)
Visual I	−0.036***	0.027*	0.095***	−0.056***	−0.016	−0.081***	0.096***	0.007
	(0.007)	(0.011)	(0.010)	(0.016)	(0.014)	(0.017)	(0.020)	(0.020)
Visual II	-0.002	0.009	−0.059***	−0.014	0.002	−0.109***	-0.011	−0.074**
	(0.009)	(0.013)	(0.012)	(0.020)	(0.018)	(0.022)	(0.025)	(0.024)
Visual Association	-0.006	0.036***	0.079***	0.021	0.053***	−0.084***	0.151***	0.077***
	(0.007)	(0.011	(0.010)	(0.016)	(0.014)	(0.017)	(0.020)	(0.020)
Limbic	0.015*	0.092***	0.098***	0.068***	0.053***	0.023	0.154***	0.089***
	(0.006)	(0.009)	(0.009)	(0.015)	(0.013)	(0.016)	(0.018)	(0.018)
Basal Ganglia	−0.022***	0.036***	0.044***	0.009	0.023.	-0.016	0.076***	0.025
	(0.006)	(0.009)	(0.009)	(0.015)	(0.013)	(0.016)	(0.018)	(0.018)
Cerebellum	0.050***	0.100***	0.025**	0.060***	0.083***	−0.063***	0.069***	0.038*
	(0.006)	(0.009)	(0.008)	(0.014)	(0.013)	(0.015)	(0.018)	(0.017)
**R-squared**	0.033***	0.025***	0.077***	0.032***	0.017***	0.021***	0.034***	0.021***

Standard errors in parentheses
Signif. codes: 0 ’***’ 0.001 ’**’ 0.01 ’*’ 0.05 ’.’ 0.1 ’ ’ 1

Note. BriefSymp: Psychological Symptoms Inventory, NegEmo: Negative Emotional Spectrum, PosEmo: Positive Emotional,Empathy: Empathy Engagement Scale, Distress: Emotional Distress Spectrum, Sociability: Sociability and Vitality Scale, Control: Self-Regulation Control Measures, Sensitivity: Sensory and Emotional Awareness Scale

Table S5: **Category-Specific Analysis of Functional Network Contributions** Regression coefficients measure the relationship between functional network assignments and absolute covariance magnitudes across behavioral categories. Each column represents a category-specific model, with coefficients indicating the relative strength of network-behavior associations. Significant positive coefficients suggest that regions within the corresponding functional network demonstrate stronger connectivity-behavior relationships for that category, thereby characterizing category-specific functional fingerprints.

**Variable**	**BriefSymp**	**NegEmo**	**PosEmo**	**Empathy**	**Distress**	**Sociability**	**Control**	**Sensitivity**
Intercept	-0.001	-0.004	0.019^{***}	0.001	-0.010	-0.009	0.002	0.004
	(0.004)	(0.005)	(0.005)	(0.008)	(0.007)	(0.009)	(0.010)	(0.010)
Eyes	-0.003	0.009	-0.006	-0.001	0.013	0.011	0.009	0.003
	(0.005)	(0.007)	(0.008)	(0.012)	(0.010)	(0.012)	(0.015)	(0.014)
GradCPT	-0.012^{*}	-0.015^{**}	-0.015^{*}	−0.020.	-0.010	0.000	-0.009	-0.021
	(0.005)	(0.007)	(0.008)	(0.012)	(0.010)	(0.012)	(0.015)	(0.014)
N-Back	-0.011^{*}	-0.007	-0.009	-0.008	0.003	0.022.	0.004	-0.008
	(0.005)	(0.007)	(0.008)	(0.012)	(0.010)	(0.012)	(0.015)	(0.014)
Stop Signal	0.008	0.023^{***}	-0.025^{***}	0.010	0.028^{**}	0.000	0.000	0.013
	(0.005)	(0.007)	(0.008)	(0.012)	(0.010)	(0.012)	(0.015)	(0.014)
Second Resting	-0.005	0.013	-0.012	-0.001	0.011	0.002	0.025.	0.002
	(0.005)	(0.007)	(0.008)	(0.012)	(0.010)	(0.012)	(0.015)	(0.014)
Average	-0.005	0.007	-0.024^{**}	-0.001	0.009	0.006	-0.007	-0.010
	(0.005)	(0.007)	(0.008)	(0.012)	(0.010)	(0.012)	(0.015)	(0.014)
**R-squared**	0.001^{***}	0.002^{***}	0.001^{*}	0.001	0.002^{*}	0.001	0.001	0.001

Standard errors in parentheses
Signif. codes: 0 ’***’ 0.001 ’**’ 0.01 ’*’ 0.05 ’.’ 0.1 ’ ’ 1

Note. BriefSymp: Psychological Symptoms Inventory, NegEmo: Negative Emotional Spectrum, PosEmo: Positive Emotional, Empathy: Empathy Engagement Scale, Distress: Emotional Distress Spectrum, Sociability: Sociability and Vitality Scale, Control: Self-Regulation Control Measures, Sensitivity: Sensory and Emotional Awareness Scale

Table S6: **Regression Analysis of Covariance Estimates Across Tasks and Behavioral Categories** Regression coefficients quantify task-specific effects on covariance estimates for each behavioral category, using the first resting-state condition (Rest1) as reference. For each behavioral category, coefficients represent the differential impact of each task condition on the magnitude of brain-behavior covariance estimates relative to Rest1. Higher absolute coefficient values indicate stronger task-specific modulation of brain-behavior relationships.

Conditions	Medial Frontal	Fronto-parietal	Default Mode	Motor	Visual I
Rest	1.938	5.875	2.063	5.188	0.188
Eyes	2.375	3.875	2.625	4.875	1.000
gradCPT	1.750	5.500	2.375	4.625	0.875
EN-Back	2.143	5.286	1.857	5.429	0.143
SST	2.000	5.500	2.375	5.250	0.375
Average	2.000	5.375	2.500	4.375	0.875

Conditions	Visual II	Visual Association	Limbic	Basal Ganglia	Cerebellum
Rest	0.000	0.813	3.813	0	0
Eyes	0.375	1.250	3.625	0	0
gradCPT	0.125	0.875	3.125	0	0
EN-Back	0.286	1.714	3.143	0	0
SST	0.125	1.000	3.375	0	0
Average	0.000	0.375	3.875	0	0

Table S7: **Distribution of Functional Biomarkers Across fMRI Conditions** Quantitative summary of functional network engagement patterns across different fMRI conditions. Values represent the mean count of functional biomarkers (from top 20 significant brain regions) associated with each functional system, averaged across all eight behavioral categories (BriefSymp, NegEmo, PosEmo, Empathy, Distress, Sociability, Control, and Sensitivity). Each row corresponds to a distinct fMRI condition, while columns represent different functional networks, providing a systematic comparison of network involvement patterns across scanning protocols.
